# Engagement in AI-Mediated Informal Digital Learning of English: An Adaptive Intelligence Perspective on Motivational, Psychological, and Technological Resources

**DOI:** 10.3390/jintelligence14100261

**Published:** 2026-10-15

**Authors:** Quan Quan, Yang Gao, Xiaochen Wang

**Affiliations:** School of Foreign Studies, Xi’an Jiaotong University, Xi’an 710049, China; gaoyang666@xjtu.edu.cn (Y.G.);

**Keywords:** AI-IDLE, adaptive intelligence, L2 motivational selves, psychological capital, L2 grit, AI literacy, learner engagement

## Abstract

Using adaptive intelligence as a theoretical lens, the study investigates how L2 motivational selves are associated with AI-IDLE engagement through psychological capital (PsyCap) and L2 grit and whether these indirect associations vary with AI literacy among 1025 Chinese university EFL learners. The proposed model was tested using structural equation modeling (SEM). For the ideal L2 self, associations with engagement emerged both directly and indirectly through PsyCap and L2 grit, whereas the ought-to L2 self showed an indirect association through PsyCap and no direct association with engagement. Higher AI literacy corresponded to stronger associations of both PsyCap and L2 grit with engagement. The two motivational self-guides were therefore associated with engagement through different psychological pathways, with AI literacy related to variation in the strength of these pathways. From an adaptive intelligence perspective, this conditional pattern indicates that the relevance of PsyCap and L2 grit to AI-IDLE engagement varies with learners’ AI literacy. These findings extend the application of adaptive intelligence to informal human–AI learning.

## 1. Introduction

The growing availability of artificial intelligence (AI) has created new possibilities for English learning beyond teacher-directed classroom activities, particularly through tools that learners can access independently for personalized support and interaction (Soyoof et al., 2023). AI-mediated informal digital learning of English (AI-IDLE) refers to learners’ self-directed use of AI for English learning beyond formal instruction (G. L. Liu et al., 2024a; X. Wang et al., 2026a). Because participation is less constrained by scheduled instruction, learners themselves make many of the decisions concerning when, how, and for what purposes AI is used (G. L. Liu et al., 2024b). Such conditions make AI-IDLE relevant to adaptive intelligence, which concerns effective responses to situational demands and the use of available resources when circumstances require adjustment and judgment (Sternberg, 2019, 2021, 2024). Within AI-IDLE, learners must maintain their own involvement while making decisions about the value of AI-generated support, the suitability of particular tools, and the ways in which technological assistance serves their learning goals (G. L. Liu et al., 2024a). Engagement in this setting can therefore reveal how learner resources relate to continued participation under changing technological conditions.

L2 self-guides may help orient learners’ motivation in this environment. The ideal L2 self concerns the language user learners hope to become, whereas the ought-to L2 self reflects attributes associated more closely with perceived expectations and obligations (Dörnyei, 2009; Papi, 2010). The ideal L2 self has shown relatively broad associations with out-of-class and self-directed language learning outcomes (J. S. Lee & Lu, 2023; You et al., 2016). Findings concerning the ought-to L2 self have been less consistent (Tahmouresi & Papi, 2021). This distinction is especially pertinent to AI-IDLE because sustained participation depends heavily on learners’ willingness to initiate and continue learning activities without continuous external regulation (Soyoof et al., 2023). The two self-guides may therefore be connected with engagement through different psychological processes.

Motivational direction alone may provide only part of this explanation. PsyCap and L2 grit capture different psychological resources relevant to goal pursuit, although much of the existing evidence has come from formal learning settings (Pawlak et al., 2022; Teimouri et al., 2022). PsyCap encompasses self-efficacy, hope, optimism, and resilience (Luthans et al., 2007), whereas L2 grit concerns continuity of effort and interest in language learning (Feng & Papi, 2020). AI literacy introduces a different dimension because it concerns learners’ competence in understanding, using, and evaluating the technological support available to them. Research in AI-supported learning has associated AI literacy with motivation and self-efficacy (C. Wang & Wang, 2025), enjoyment (H. Liu & Fan, 2025), and engagement (X. Wang et al., 2025). Bringing these constructs together makes it possible to examine whether the associations of PsyCap and L2 grit with engagement differ according to learners’ AI literacy. This conditional question is also relevant to adaptive intelligence because it concerns whether the role of personal resources varies with competence suited to the environment in which those resources are used.

Previous AI-IDLE research has examined motivational self-guides and related affective processes (X. Wang et al., 2026b), psychological resources (Quan et al., 2025), and AI-related competence (Xu, 2025). It remains unclear whether, within the same model, the ideal L2 self and ought-to L2 self are differently associated with engagement through PsyCap and L2 grit and whether these pathways vary across levels of AI literacy. Accordingly, the present study tests a moderated mediation model among Chinese university EFL learners. The model includes direct relationships of the ideal L2 self and ought-to L2 self with AI-IDLE engagement, indirect relationships through PsyCap and L2 grit, and moderation by AI literacy on the resource-to-engagement relationships. For AI-IDLE research, this design clarifies how motivational orientation, psychological resources, and technological competence are related within one explanatory model. For adaptive intelligence research, it offers evidence from a self-directed human–AI learning environment in which the associations between personal resources and engagement can be examined in relation to context-specific technological competence.

## 2. Literature Review

### 2.1. AI-Mediated Informal Digital Learning of English

AI-IDLE builds on the broader literature on informal digital learning of English, which examines learner-initiated English learning through digital resources beyond formal instruction (J. S. Lee & Lee, 2021; Soyoof et al., 2023). G. L. Liu et al. (2024a) extended this line of work to AI-supported settings by conceptualizing AI-IDLE around learners’ autonomous use of AI for self-directed English learning beyond classroom instruction. Such learning can involve feedback, personalized resources, language practice, and other forms of interaction selected and managed by learners themselves.

Recent AI-IDLE research has examined a growing range of learner characteristics associated with continued participation. Motivational self-guides and affective experiences have received increasing attention in AI-IDLE research, including work on enjoyment and other achievement emotions (G. L. Liu et al., 2024b; X. Wang et al., 2026b). Other studies have examined resilience and self-efficacy in AI-mediated learning (Quan et al., 2025), while writing motivation and enjoyment have also been investigated in AI-IDLE (Zou et al., 2025). Related research on informal digital learning has also identified self-efficacy, digital learning competence, and metacognitive regulation as relevant to learners’ involvement outside the classroom (Fang et al., 2026; Ko, 2026). This body of research indicates that participation in digital learning reflects both access to technology and learners’ motivational, psychological, and self-regulatory resources.

AI-supported informal learning also differs from many conventional learning settings because decisions about technological support are largely made by learners. They must determine which tools are useful, how generated information should be interpreted, and how technological assistance fits their current learning purposes. Research on AI-assisted learning has similarly emphasized self-regulation, higher-order thinking, and active knowledge construction in productive technology use (H. Y. Lee et al., 2024). AI-IDLE therefore brings learners’ personal resources into close relation with their competence in using technology.

Although existing work has identified a range of motivational, psychological, and technological correlates of AI-IDLE, less is known about how these elements operate together. In particular, the processes linking different motivational self-guides with engagement through distinct psychological resources, and the extent to which these processes vary with learners’ AI-related competence, remain insufficiently clarified.

### 2.2. L2 Motivational Self-Guides

The ideal L2 self and ought-to L2 self are two central components of the L2 Motivational Self System proposed by Dörnyei (2009). The framework explains language learning motivation partly through learners’ images of themselves as future language users and originally includes the ideal L2 self, the ought-to L2 self, and the L2 learning experience. Later work has further differentiated future self-guides according to their origins and regulatory characteristics, showing that different self-guides can be associated with different forms of motivated behavior (Papi et al., 2019). This development makes it important to examine the ideal L2 self and ought-to L2 self separately as distinct components of future-oriented motivation.

The ideal L2 self refers to the language-related attributes learners personally hope to possess in the future. It incorporates internally valued aspirations and motivates behavior directed toward reducing the gap between learners’ present and desired future selves (Dörnyei, 2009; Papi, 2010). The ought-to L2 self, by comparison, concerns qualities learners believe they should develop in response to perceived expectations, duties, or possible negative consequences (Dörnyei, 2009).

Evidence from language learning has generally shown a broader role for the ideal L2 self in voluntary and self-directed behavior. It has been associated with out-of-class digital learning, willingness to communicate, and positive emotional experiences (J. S. Lee & Lu, 2023; You et al., 2016). Associations involving the ought-to L2 self have tended to be weaker in autonomous extramural learning, although some relationships with positive emotional experiences have been reported (Tahmouresi & Papi, 2021; Yung & Chiu, 2023). Recent AI-IDLE research has also identified different patterns for these two self-guides, with the ideal L2 self showing wider links with achievement emotions and engagement (X. Wang et al., 2026b). This distinction may become especially important in AI-mediated learning because learners can pursue personally meaningful goals with considerable autonomy, whereas externally driven motives may be more closely related to fulfilling immediate task requirements (Song & Song, 2023).

The two self-guides may therefore relate to engagement through different psychological processes. Examining PsyCap and L2 grit in parallel provides a way to distinguish between resources involved in responding to learning challenges and those involved in maintaining longer-term goal pursuit.

### 2.3. Psychological Capital and L2 Grit as Distinct Psychological Resources

PsyCap brings together self-efficacy, hope, optimism, and resilience within a higher-order psychological construct (Luthans et al., 2007). These components reflect positive psychological resources that can be drawn upon during goal pursuit, particularly when individuals encounter setbacks, uncertainty, or demanding tasks. Educational research has also associated PsyCap with motivation, engagement, and sustained involvement in learning (Datu et al., 2018; Li et al., 2023). Within AI-supported EFL learning, stronger PsyCap has likewise been associated with greater engagement (Derakhshan & Azari Noughabi, 2024), indicating that PsyCap may be particularly relevant in technologically mediated learning settings.

L2 grit captures a different aspect of sustained goal pursuit. Teimouri et al. (2022) define L2 grit through perseverance of effort and consistency of interest specifically in language learning. Feng and Papi (2020) connected L2 grit with persistence, while subsequent studies have examined its associations with motivated behavior and engagement (Pawlak et al., 2022; Zhao et al., 2026). Its relevance may be particularly pronounced when learning depends on learners’ own willingness to continue despite difficulty, as in online and other self-directed settings (M. Wang & Zhang, 2026). Studies have also linked grit with classroom and online engagement through relationships involving enjoyment and self-efficacy (Derakhshan & Fathi, 2023; Jin, 2024). In informal digital learning specifically, L2 grit has been examined together with self-efficacy and digital literacy as a contributor to learners’ participation (Barkati et al., 2025).

Although PsyCap and L2 grit both concern successful goal pursuit, they emphasize different psychological functions. PsyCap is more closely connected with the resources learners can draw on when responding to challenges, whereas L2 grit concerns the continuity of effort and interest across time. AI-IDLE can place demands on both. Learners may need psychological resources to manage difficulties that arise during AI use while also maintaining participation without continuous external supervision. Treating PsyCap and L2 grit as parallel pathways therefore allows their distinct roles in linking motivation with engagement to be examined.

### 2.4. L2 Engagement

Learner engagement is commonly conceptualized as a multidimensional construct involving behavioral, cognitive, affective, and social participation (Hiver et al., 2024; Y. Wang & Xue, 2024; Zhou et al., 2021). Its expression also varies across tasks, activities, and learning environments (Hiver et al., 2024). This contextual character is particularly important for AI-IDLE because participation occurs outside scheduled instruction and often relies on learners’ own decisions to begin, continue, and regulate learning activity (X. Wang et al., 2026a).

In AI-supported EFL learning, engagement develops within an environment that combines considerable learner autonomy with cognitive and emotional demands. Under such conditions, personal psychological resources may become especially relevant to continued involvement (Huang & Liang, 2026). Existing studies have examined AI-mediated engagement in relation to motivational self-guides (X. Wang et al., 2026b), resilience and AI literacy (X. Wang et al., 2025), and characteristics of the learning environment (Y. Wang & Xue, 2024). These findings suggest that engagement reflects the joint relevance of learner characteristics and the conditions surrounding AI use. It therefore serves as an appropriate outcome for examining how motivational, psychological, and technological factors come together in AI-IDLE.

### 2.5. AI Literacy as a Potential Moderator

As GenAI becomes increasingly embedded in everyday learning, AI literacy can be viewed as an AI-specific extension of broader digital competence (Ng et al., 2021). Ng et al. (2024) organize AI literacy around four components. Two concern knowing and understanding AI and using and applying AI, while the other two address evaluating AI-generated content and engaging with AI in ethical ways. This view places critical judgment alongside technical knowledge and use. More recent conceptual work similarly distinguishes basic knowledge of AI from the ability to apply that knowledge appropriately in particular situations, with evaluation and responsible use occupying a central role (Chiu, 2025). These capacities are especially relevant in informal settings because learners receive less direct guidance about when AI should be used or how its output should influence subsequent learning.

Empirical work increasingly links AI literacy with learners’ experiences in AI-supported education. Higher AI literacy has been associated with stronger self-efficacy and motivation (C. Wang & Wang, 2025), greater enjoyment (H. Liu & Fan, 2025), and more active engagement with AI-mediated learning (Quan et al., 2025). Among Chinese university language learners, individual dimensions of AI literacy have also shown different relationships with self-directed learning, indicating that the ability to understand and critically evaluate AI may affect how learners manage technology-supported activities (Xu, 2025). Studies in EFL classrooms have also reported relationships among AI literacy, resilience, and engagement (X. Wang et al., 2025).

This combination is particularly relevant to AI-IDLE. Psychological resources may help learners persist or respond to difficulties, yet participation also requires sufficient competence to judge, select, and use technological support. AI literacy may therefore alter how strongly PsyCap and L2 grit are associated with engagement, making it a plausible contextual condition within the proposed model.

### 2.6. Theoretical Framework

SDT provides the motivational basis for distinguishing the two L2 self-guides examined in this study (Deci & Ryan, 1985, 2000). A central principle of SDT is that motivation differs in the degree to which behavior is internally endorsed, spanning externally regulated behavior and more autonomous forms grounded in personal interests and values (Ryan & Deci, 2000). Research in second language learning has similarly shown that differences in motivational orientation are related to learners’ effort and learning outcomes (Alamer, 2022). Applied to L2 self-guides, this perspective helps explain why personally valued future aspirations and externally grounded obligations may show different relationships with psychological resources and engagement. The ideal L2 self is more closely rooted in personally valued future goals, whereas the ought-to L2 self is more strongly shaped by expectations and obligations. Previous studies have generally reported broader associations between the ideal L2 self and autonomous learning outcomes (Feng & Papi, 2020; Papi et al., 2019), whereas research in out-of-class and AI-IDLE settings has indicated a more limited role for the ought-to L2 self (Tahmouresi & Papi, 2021; X. Wang et al., 2026b).

The ideal L2 self can provide direction for continued goal pursuit and may be associated with confidence, responses to difficulty, and sustained effort. The ought-to L2 self can also guide behavior when learners seek to satisfy external expectations or avoid undesirable consequences. On this basis, both self-guides are expected to relate positively to the psychological resources and engagement examined here, with the ideal L2 self expected to show the broader pattern in self-directed AI-mediated learning.

**H1a.**  *The ideal L2 self is positively associated with PsyCap, L2 grit, and AI-IDLE engagement.*

**H1b.**  *The ought-to L2 self is positively associated with PsyCap, L2 grit, and AI-IDLE engagement.*

PsyCap and L2 grit are positioned as parallel psychological pathways because they capture different aspects of goal pursuit. PsyCap concerns resources relevant to coping with challenges and maintaining goal-directed functioning, whereas L2 grit reflects continued effort and interest over a longer period. Both have previously been associated with persistence and engagement in language learning and digitally mediated environments (Barkati et al., 2025; Derakhshan & Azari Noughabi, 2024; Derakhshan & Fathi, 2023). These functions are particularly pertinent to AI-IDLE, where learners must handle difficulties arising during AI-supported activity while sustaining participation with relatively little continuous external regulation.

**H2.**  *PsyCap and L2 grit are positively associated with AI-IDLE engagement.*

**H3a.**  *PsyCap positively mediates the associations between the ideal and ought-to L2 selves and AI-IDLE engagement.*

**H3b.**  *L2 grit positively mediates the associations between the ideal and ought-to L2 selves and AI-IDLE engagement.*

AI literacy is introduced as a contextual condition that may alter the relationship between these psychological resources and engagement. Learners with stronger AI literacy are better positioned to understand what AI systems can provide, assess the quality of their output, and choose forms of support that suit particular learning purposes. Such competence may change how psychological resources such as confidence, resilience, and persistence relate to actual participation in AI-mediated learning. Prior evidence connecting AI literacy with both engagement and psychological resources provides support for examining this conditional relationship (H. Liu & Fan, 2025; X. Wang et al., 2025; Xu, 2025).

**H4a.**  *AI literacy positively moderates the association between PsyCap and AI-IDLE engagement, such that the association is stronger at higher levels of AI literacy.*

**H4b.**  *AI literacy positively moderates the association between L2 grit and AI-IDLE engagement, such that the association is stronger at higher levels of AI literacy.*

**Figure 1:**
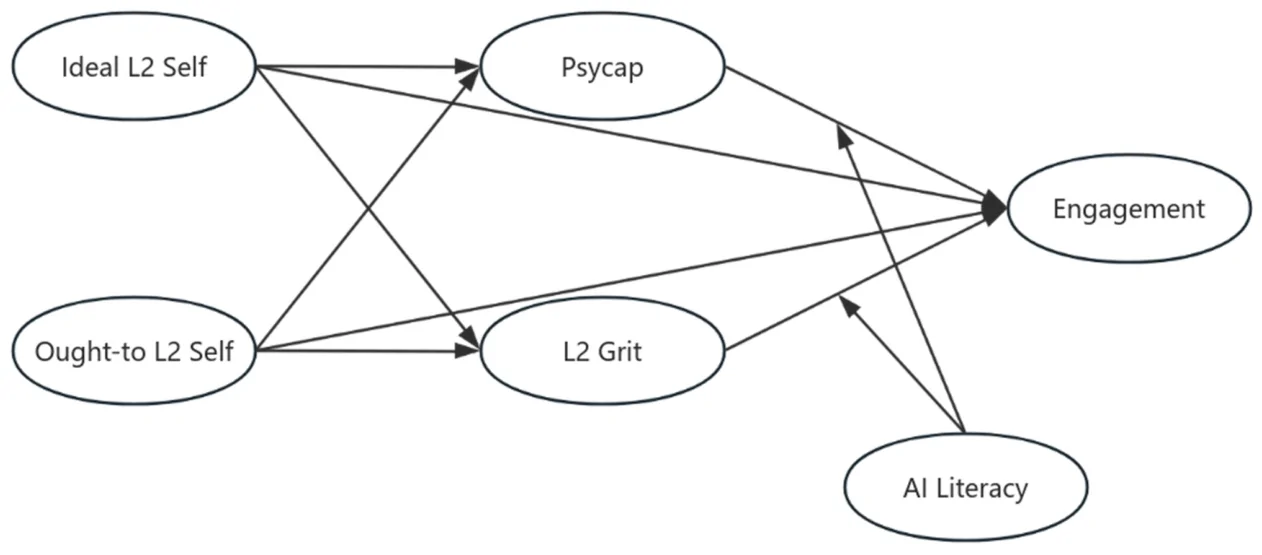
The proposed model.

These conditional relationships also provide the main connection between the proposed model and adaptive intelligence. Adaptive intelligence emphasizes how individuals use available resources when responding to changing task and environmental requirements (Sternberg, 2019, 2021, 2024; Sternberg et al., 2022). Within the present model, motivational self-guides provide direction, PsyCap and L2 grit represent resources relevant to goal pursuit, and AI literacy captures competence specific to the technological setting. Examining whether the indirect relationships between motivational self-guides and engagement vary across levels of AI literacy therefore addresses a question central to adaptive intelligence, namely whether the relevance of personal resources changes with competence suited to the environment in which those resources are used. The proposed model is presented in Figure 1.

**H5.**  *The indirect associations of the ideal and ought-to L2 selves with AI-IDLE engagement through PsyCap and L2 grit are stronger at higher levels of AI literacy.*

## 3. Method

### 3.1. Participants

Participants were eligible if they were university EFL learners studying in mainland China and had previously used AI tools for English learning beyond formal classroom instruction. Recruitment was conducted through open online channels on WeChat and Xiaohongshu using convenience sampling. Because institutional affiliation was not collected, the exact number and types of universities represented in the sample could not be determined. Data collection took place between 17 March and 9 April 2026. Of the 1223 questionnaires submitted, responses were removed when the attention-check item was answered incorrectly or when completion time was shorter than 60 s. This screening procedure resulted in a final sample of 1025 participants.

Prior to data collection, ethical approval was granted by the Ethics Committee of the School of Foreign Studies, Xi’an Jiaotong University (Approval No. XJTUSFREA[2024]-1-001). The study followed the APA Ethical Principles and the Declaration of Helsinki. Before beginning the questionnaire, participants provided electronic informed consent. Participation was voluntary, withdrawal was permitted at any point, and no personally identifying information was collected in the responses. Participant characteristics are summarized in Table 1.

### 3.2. Instruments

The survey was presented in Chinese and included demographic questions followed by the scales used to assess the study constructs. Previously developed and validated instruments were used throughout, with wording adjusted to the present context where necessary. The Chinese version was prepared through translation and back-translation. Two specialists in applied linguistics and foreign language education compared the original, translated, and back-translated versions with attention to semantic equivalence and suitability for the study context. Any discrepancies identified during this process were resolved before data collection. Responses to all scale items were recorded on a five-point Likert scale from 1 = strongly disagree to 5 = strongly agree. Reverse-keyed items were recoded before the analyses.

#### 3.2.1. Ideal L2 Self and Ought-To L2 Self

The two motivational self-guides were assessed with the scales reported by You et al. (2016), validated in the Chinese EFL context. Five items assessed the ideal L2 self by capturing learners’ envisioned future selves as proficient English users. Six items assessed the ought-to L2 self by focusing on language-related attributes learners perceived that they should develop in response to external expectations. Each self-guide was treated as a separate first-order latent construct. For the ideal L2 self, a one-factor CFA produced satisfactory fit, χ^2^/df = 0.919, GFI = 0.998, NFI = 0.998, and SRMR = 0.008. The corresponding one-factor model for the ought-to L2 self also fitted the data well, χ^2^/df = 1.754, CFI = 0.998, TLI = 0.997, RMSEA = 0.027, and SRMR = 0.013.

#### 3.2.2. Psychological Capital

PsyCap was assessed with the 16-item instrument developed by King and Caleon (2021). Its four dimensions comprise self-efficacy, hope, optimism, and resilience, with four items representing each dimension. Because PsyCap is conceptualized as a state-like psychological resource that can vary across contexts, the items were adapted to refer specifically to AI-mediated informal English learning outside the classroom. Its measurement structure was examined through a second-order CFA in which the four dimensions served as first-order factors loading on the overall PsyCap construct. Fit indices supported this specification, χ^2^/df = 1.535, CFI = 0.994, TLI = 0.993, RMSEA = 0.023, and SRMR = 0.023.

#### 3.2.3. L2 Grit

L2 grit was assessed with the nine-item scale developed by Teimouri et al. (2022), covering perseverance of effort and consistency of interest in language learning. No AI-IDLE-specific changes were made to the wording of this instrument. Negatively phrased items were reverse scored before analysis. Consistent with the measurement specification used in the present study, all nine items loaded on a single overall L2 grit factor. The one-factor CFA yielded satisfactory fit, χ^2^/df = 1.913, CFI = 0.996, TLI = 0.995, RMSEA = 0.030, and SRMR = 0.014.

#### 3.2.4. Engagement

Engagement in AI-IDLE was assessed using 16 items adapted from Su and Guo (2021). The instrument covers behavioral, cognitive, affective, and social engagement, with four items assigned to each dimension. Wording was adjusted so that the items referred to voluntary AI-mediated English learning outside formal classroom activities. A second-order CFA was specified with the four engagement dimensions loading on an overall engagement factor. The model showed good fit, χ^2^/df = 1.270, CFI = 0.997, TLI = 0.996, RMSEA = 0.016, and SRMR = 0.021.

#### 3.2.5. AI Literacy

AI literacy was measured using the 12-item scale developed by B. Wang et al. (2023). The scale comprises four dimensions of awareness, usage, evaluation, and ethics, with three items in each dimension. Item wording was minimally contextualized to English learning while retaining the content of the original scale. A second-order CFA was specified with the four dimensions loading on an overall AI literacy factor. The model showed good fit, χ^2^/df = 1.530, CFI = 0.995, TLI = 0.994, RMSEA = 0.023, and SRMR = 0.025.

### 3.3. Data Analysis

SPSS 26 was used for descriptive statistics, Pearson correlations, and internal consistency estimates. Structural analyses were conducted in Mplus 8.3, while R 4.5.3 was used to calculate the heterotrait–monotrait ratio of correlations (HTMT). Potential common method bias was examined in two ways. Harman’s single-factor test was first conducted, followed by a CFA in which all questionnaire items were constrained to load on a single latent factor.

The measurement structure was then examined at the item level through CFA using ML estimation. The ideal L2 self, ought-to L2 self, and L2 grit were represented as first-order latent constructs. PsyCap, AI literacy, and engagement were specified as second-order constructs based on their respective first-order dimensions. Model adequacy was assessed with χ^2^/df, CFI, TLI, RMSEA, and SRMR. Reliability and construct validity were evaluated using Cronbach’s α, composite reliability, average variance extracted, the Fornell–Larcker criterion, and HTMT.

Following confirmation of the measurement properties, the moderated mediation analysis was conducted using composite scores. Using composite scores kept the interaction model more parsimonious when higher-order constructs and product terms were estimated together. PsyCap, L2 grit, and AI literacy were mean-centered prior to forming the PsyCap × AI literacy and L2 grit × AI literacy interaction terms. ML estimation was retained for the path model, with 5000 bias-corrected bootstrap resamples used for inference. Indirect and conditional indirect associations were evaluated from 95% bias-corrected bootstrap confidence intervals. Moderation was probed at the mean of AI literacy and at one standard deviation below and above the mean. Each conditional indirect pathway through PsyCap and L2 grit was estimated directly, irrespective of whether an individual component path reached statistical significance.

## 4. Results

### 4.1. Results of Common Method Bias

Harman’s single-factor test indicated that the first unrotated factor accounted for 23.243% of the total variance. A single-factor CFA was then fitted as an additional check, with all questionnaire items specified on one latent factor. As reported in Table 2, this model showed poor fit and was substantially inferior to the hypothesized measurement model. The two diagnostics therefore provided no evidence that a single common factor adequately represented the covariance among the measured variables.

### 4.2. Measurement Model

The measurement model estimated with ML yielded satisfactory fit according to the recommended criteria (Kline, 2023), with the fit indices reported in Table 2. Standardized factor loadings ranged from 0.709 to 0.850, with all values above the 0.70 criterion (Hair et al., 2019). As reported in Table 3, CR exceeded 0.70 and AVE exceeded 0.50 (Hair et al., 2019), meeting the recommended requirements for convergent validity. Under the Fornell–Larcker criterion, the square root of AVE for each construct was greater than its correlations with every other construct (Fornell & Larcker, 1981). HTMT provided an additional assessment of discriminant validity. All values in Table 4 were below the 0.85 criterion (Henseler et al., 2015), indicating adequate empirical separation among the constructs.

### 4.3. Descriptive Statistics and Correlation Analysis

Table 5 summarizes the descriptive statistics and bivariate correlations. Skewness and kurtosis for all variables remained within ±1, supporting approximate normality. The ideal L2 self had the highest mean score (M = 3.814, SD = 0.580), whereas L2 grit had the lowest (M = 3.339, SD = 0.558). All study variables were positively correlated with engagement. PsyCap showed the strongest correlation with engagement (r = 0.530, *p* < .001), followed by the ideal L2 self (r = 0.399, *p* < .001), L2 grit (r = 0.365, *p* < .001), AI literacy (r = 0.338, *p* < .001), and the ought-to L2 self (r = 0.240, *p* < .001).

### 4.4. Direct and Indirect Associations

**Figure 2:**
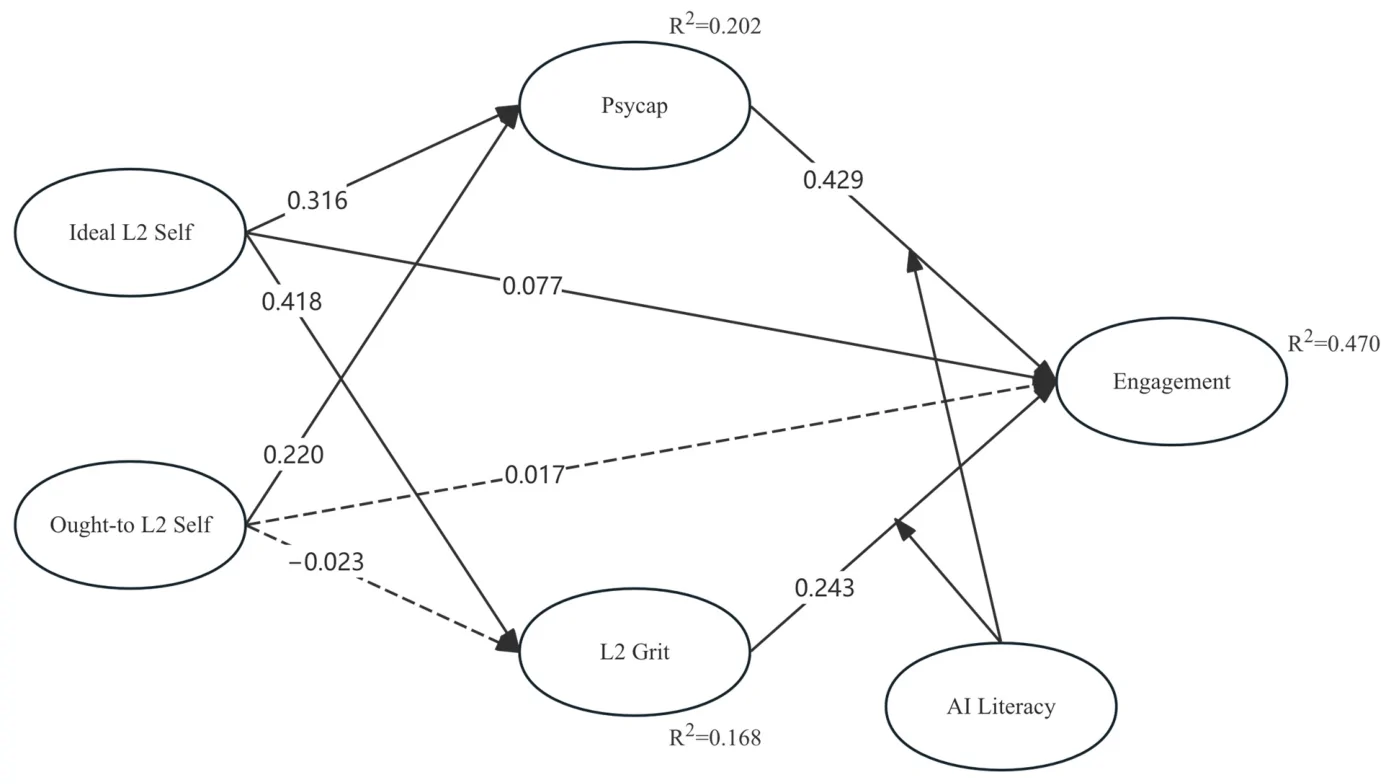
Final structural model.

Fit indices for the structural model were within the recommended ranges (Kline, 2023), as reported in Table 2. Explained variance was 47.0% for AI-IDLE engagement, 20.2% for PsyCap, and 16.8% for L2 grit. Direct and indirect estimates are reported in Table 6 and Table 7, and Figure 2 displays the final structural model.

Positive paths were obtained from the ideal L2 self to PsyCap, L2 grit, and AI-IDLE engagement, supporting H1a. For the ought-to L2 self, only the path to PsyCap was positive, whereas the paths to L2 grit and engagement were statistically indistinguishable from zero. H1b was therefore partially supported.

Positive paths from both PsyCap and L2 grit to engagement supported H2. For the indirect pathways, both the ideal L2 self and ought-to L2 self showed positive associations with engagement through PsyCap, with bootstrap confidence intervals excluding zero. This supported H3a. Through L2 grit, the indirect association was positive for the ideal L2 self, whereas the corresponding association for the ought-to L2 self included zero in its confidence interval. H3b was therefore partially supported.

### 4.5. Moderation of Psychological Resource Associations

**Figure 3:**
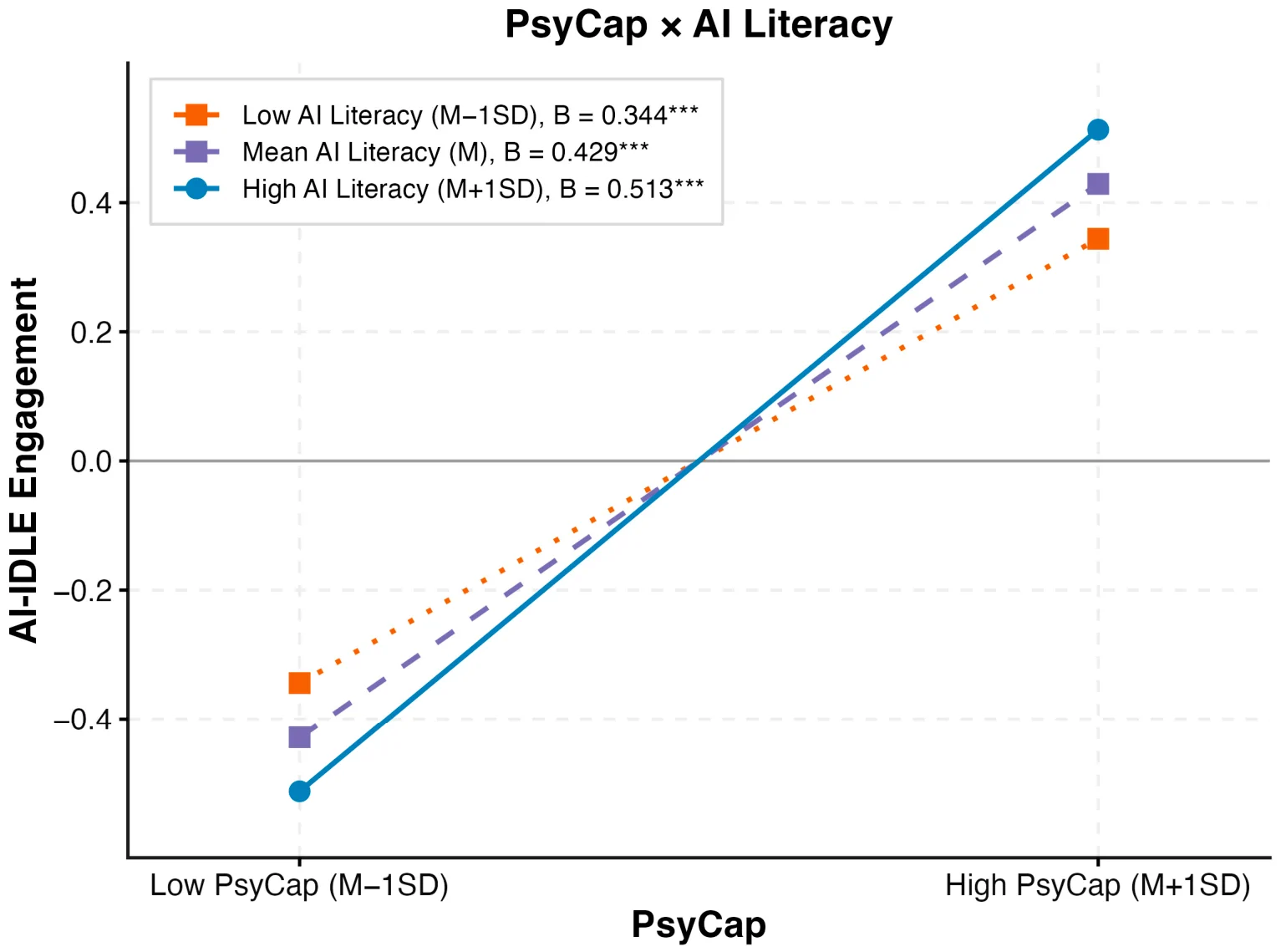
Moderating effects of AI literacy on the relationship between PsyCap and AI-IDLE engagement. Note. *** *p* < .001.

**Figure 4:**
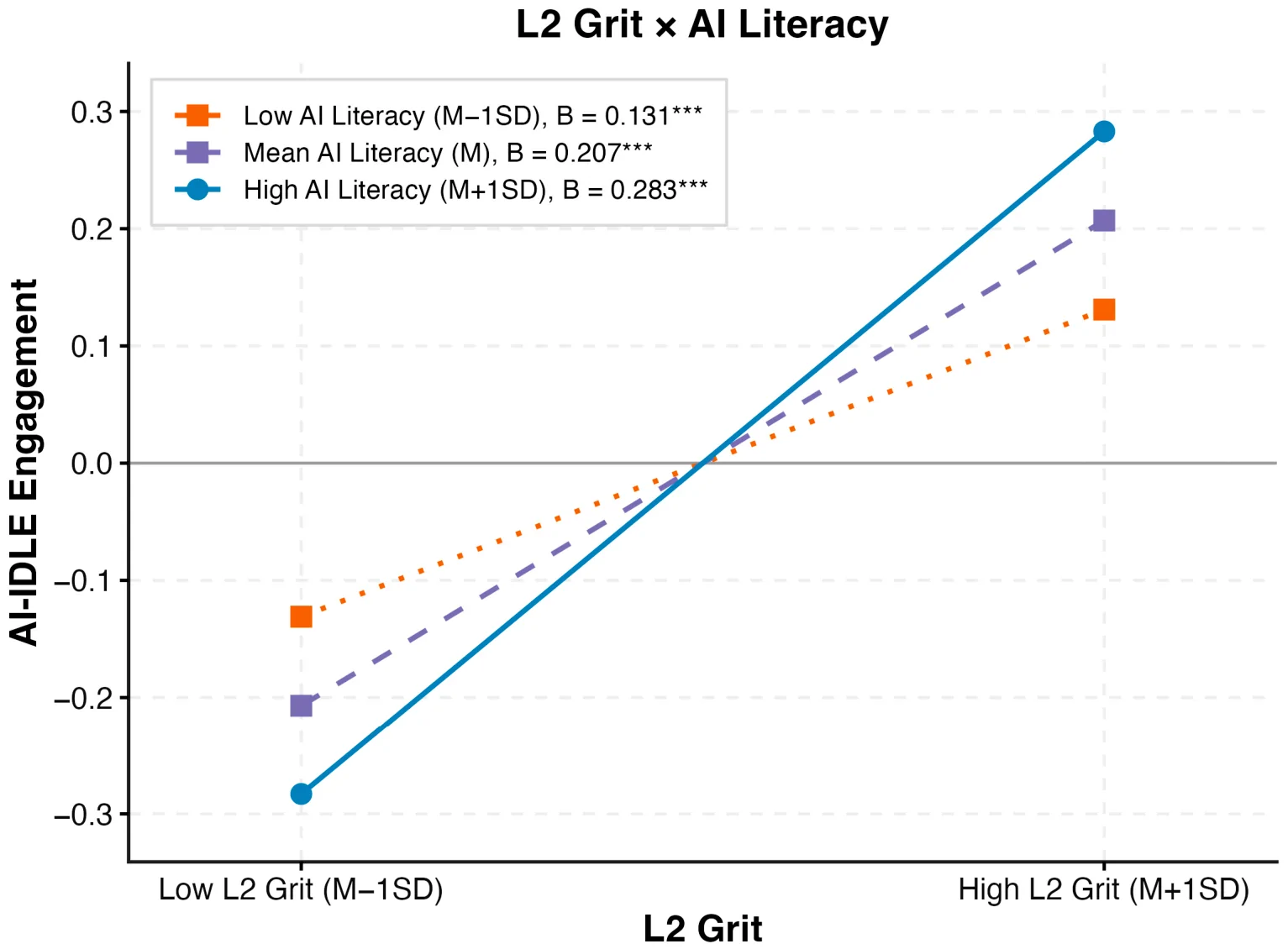
Moderating effects of AI literacy on the relationship between L2 Grit and AI-IDLE engagement. Note. *** *p* < .001.

The moderating effects of AI literacy were examined through the PsyCap × AI literacy and L2 grit × AI literacy interaction terms. Results are presented in Table 8 and Figure 3 and Figure 4.

Both interaction effects were positive. The association between PsyCap and engagement increased as AI literacy increased, supporting H4a. The association between L2 grit and engagement showed the same pattern, supporting H4b.

### 4.6. Conditional Indirect Associations

Conditional indirect associations were examined at three values of AI literacy, corresponding to one standard deviation below the mean, the mean, and one standard deviation above the mean. Variation in these associations was evaluated with the index of moderated mediation. Results are reported in Table 9.

The conditional indirect associations of both the ideal and ought-to L2 selves with engagement through PsyCap increased across levels of AI literacy. Their indices of moderated mediation were positive, with the corresponding confidence intervals excluding zero. The conditional indirect association of the ideal L2 self through L2 grit showed a similar pattern. In contrast, the conditional indirect association of the ought-to L2 self through L2 grit remained statistically indistinguishable from zero at low, mean, and high levels of AI literacy, and its index of moderated mediation also included zero. H5 therefore received partial support. These conditional patterns are consistent with an adaptive intelligence perspective, in which the contribution of personal resources depends partly on their fit with the demands of a particular environment.

## 5. Discussion

### 5.1. L2 Motivational Selves, Psychological Resources, and AI-IDLE Engagement

The pattern observed for the ideal L2 self aligns with prior findings on the motivational breadth of internalized self-guides in language learning (Fathi & Hejazi, 2023; X. Wang et al., 2026b). The ideal L2 self may carry particular relevance in AI-IDLE because learners assume substantial responsibility for initiating learning activities, dealing with difficulties, and maintaining their involvement beyond formal classroom requirements. Within this process, PsyCap and L2 grit may serve complementary psychological functions. PsyCap is relevant to learners’ responses when challenges emerge in goal pursuit, whereas L2 grit concerns maintaining effort and interest across time (Luthans et al., 2007; Teimouri et al., 2022). Accordingly, the ideal L2 self may relate to AI-IDLE engagement through learners’ responses to immediate challenges and their continued pursuit of longer-term language goals.

The narrower pattern for the ought-to L2 self may reflect its greater dependence on external expectations. Academic, social, and family expectations can still provide direction for language learning, although their relevance to sustained personal interest in voluntary learning may be weaker. Feng and Papi (2020) likewise reported a relationship between the ought-to L2 self and motivational intensity, alongside a weaker relationship with perseverance. Because L2 grit entails sustained effort and consistency of interest across time (Teimouri et al., 2022), the weaker association between the ought-to L2 self and L2 grit may indicate that externally grounded obligations are less relevant to persistence in voluntary learning. X. Wang et al. (2026b) similarly reported a relatively limited role for the ought-to L2 self in autonomous out-of-class learning.

The contrast therefore suggests different psychological pathways from the ideal L2 self and ought-to L2 self to AI-IDLE engagement. Among the Chinese university EFL learners examined here, the ideal L2 self appears particularly relevant when learners themselves must sustain participation. This pattern accords with the SDT emphasis on internalized motivation in self-directed behavior. It also supports distinguishing the functions of PsyCap and L2 grit within the motivational process.

### 5.2. AI Literacy, Conditional Associations, and Adaptive Functioning

AI literacy adds an important contextual dimension to the role of psychological resources in AI-IDLE. Zhang et al. (2025) and Zheng (2026) have examined AI literacy in relation to learner engagement or motivation. The present study extends this line of research by suggesting that the relevance of psychological resources to engagement may also depend on learners’ competence within the technological environment where these resources operate.

This interpretation is pertinent to AI-IDLE, where learners themselves decide which tools to use, how to evaluate AI-generated information, and how technological support should contribute to their learning goals. The associations of PsyCap and L2 grit with engagement may therefore be stronger when learners also have sufficient AI literacy to evaluate and use AI effectively.

AI literacy may also play different roles across motivational processes. AI literacy may support learners in applying psychological resources during self-directed activity, while its relevance may be more limited when the underlying motivational orientation is less closely connected with sustained personal interest. This distinction helps explain why AI literacy may accompany different motivational pathways without operating uniformly across them. Zhang et al. (2025) reported a related classroom pattern in which AI literacy was indirectly associated with willingness to communicate through self-efficacy. Together, these findings suggest that the role of AI literacy may depend partly on the learning context and the degree of learner responsibility for selecting, evaluating, and using AI tools.

These relationships also offer a basis for interpreting the findings through adaptive intelligence. Adaptive intelligence emphasizes effective responses to task and environmental demands through the use and coordination of available personal resources (Sternberg, 2019, 2021, 2024). AI-IDLE presents a particularly relevant setting because learners must pursue language goals while adapting to a technological environment that requires continual judgment about how AI should be selected, evaluated, and used. From this perspective, PsyCap and L2 grit matter as learner resources whose relevance to engagement varies with competence specific to the environment. The conditional role of AI literacy therefore illustrates a central feature of adaptive intelligence, namely that the contribution of personal resources is context-dependent and shaped by the demands under which those resources are used.

The evaluative component of AI literacy is especially pertinent here, as learners need to judge AI systems’ capabilities and limitations and determine their appropriate use for particular learning purposes. From an adaptive intelligence perspective, such evaluative processes are relevant because effective adaptation involves selecting and using available resources in response to task and environmental demands (Sternberg, 2019, 2021, 2024). In AI-IDLE, these processes are embedded in practical decisions about evaluating AI-generated support, adjusting learning strategies, and pursuing learning goals with limited external guidance.

### 5.3. Pedagogical Implications

The findings suggest that AI literacy may warrant attention when universities support students’ engagement in AI-mediated informal language learning. Given that the associations of PsyCap and L2 grit with engagement were stronger at higher levels of AI literacy, EFL courses may provide opportunities for learners to develop a more informed understanding of how AI tools can be used for language learning. Such opportunities may include examining the capabilities and limitations of common AI tools, evaluating the accuracy and potential biases of AI-generated content, and considering privacy and ethical issues associated with AI use (Ng et al., 2024; B. Wang et al., 2023).

Equitable access should also be considered when AI-supported learning is encouraged. Learners may differ in their access to advanced AI services, suitable devices, stable internet connections, and opportunities to develop AI-related competence. Such differences may shape participation in AI-IDLE and may widen existing inequalities in learning opportunities. Universities may therefore consider providing accessible technological resources and alternative forms of support where feasible.

These considerations highlight the importance of attending to AI literacy and the conditions surrounding learners’ access to and use of AI. In the Chinese university EFL context examined here, the findings suggest that AI literacy and equitable access are relevant considerations when supporting AI-mediated informal learning.

## 6. Conclusions

Among the Chinese university EFL learners examined here, the ideal L2 self, ought-to L2 self, PsyCap, L2 grit, and AI literacy were related to AI-IDLE engagement in different ways. The ideal L2 self was associated with engagement both directly and indirectly through PsyCap and L2 grit, whereas the ought-to L2 self showed an indirect association through PsyCap, with no corresponding indirect association through L2 grit. The associations of PsyCap and L2 grit with AI-IDLE engagement also became stronger at higher levels of AI literacy. Viewed through adaptive intelligence, these conditional patterns suggest that the relevance of PsyCap and L2 grit to AI-IDLE engagement differs across levels of learners’ AI literacy.

Several limitations of this study should be acknowledged. The use of self-report measures and a variable-centered analytic design may capture only part of how learners select AI tools, formulate and revise prompts, evaluate AI feedback, and adjust strategies during specific AI-IDLE episodes. In addition, the AI literacy measure captures general competence, while tool-specific prompting practices and forms of critical evaluation tied to particular AI systems warrant separate investigation. The exclusive focus on Chinese university EFL learners narrows the range of cultural and institutional contexts to which the findings can be applied. The cross-sectional design also means that the temporal sequence and causal direction of the relationships cannot be established. Future research could combine behavioral logs, screen recordings, experience sampling, and qualitative process data to capture AI-IDLE more directly. Longitudinal or experimental designs could further examine how the ideal L2 self, ought-to L2 self, PsyCap, L2 grit, and AI literacy develop and relate to AI-IDLE engagement over time.

## Figures and Tables

**Table 1:** Demographic information.

	Item	Number	Percentage (%)
Gender	Male	511	49.85
	Female	514	50.15
Age	18–20	267	26.05
	21–22	326	31.8
	23–25	320	31.22
	More than 25	112	10.93
Major	Humanities and Social Sciences	271	26.44
	Science, Technology, Engineering, and Mathematics	291	28.39
	Arts and Physical Education	128	12.49
	Medicine and Agriculture	106	10.34
	Business and Management	229	22.34

**Table 2:** Model fit indices.

	χ^2^/df	RMSEA	CFI	TLI	SRMR
Single-factor model	13.359	0.110	0.367	0.346	0.134
Measurement model	1.495	0.022	0.975	0.974	0.042
Structural model	1.591	0.024	0.997	0.990	0.018
Cutoff value (Kline, 2023)	<5	<0.10	>0.90	>0.90	<0.08

**Table 3:** Construct reliability and validity.

	α	CR	AVE	1	2	3	4	5	6
1. Ideal L2 Self	0.889	0.889	0.615	**0.784**					
2. Ought-to L2 Self	0.907	0.907	0.621	0.433	**0.788**				
3. PsyCap	0.925	0.963	0.633	0.476	0.401	**0.796**			
4. L2 Grit	0.935	0.935	0.614	0.449	0.155	0.206	**0.784**		
5. AI Literacy	0.892	0.951	0.592	0.156	0.066	0.075	0.061	**0.769**	
6. Engagement	0.925	0.962	0.637	0.472	0.287	0.651	0.419	0.428	**0.798**

Note: (1) PsyCap = Psychological Capital; (2) the square roots of AVE are shown in bold on the diagonal.

**Table 4:** HTMT ratios.

	1	2	3	4	5	6
1. Ideal L2 Self	1					
2. Ought-to L2 Self	0.433	1				
3. PsyCap	0.451	0.380	1			
4. L2 Grit	0.449	0.155	0.195	1		
5. AI Literacy	0.147	0.063	0.067	0.057	1	
6. Engagement	0.447	0.272	0.584	0.397	0.383	1

Note: PsyCap = Psychological Capital.

**Table 5:** Results of descriptive statistics and correlation analysis.

	1	2	3	4	5	6
1. Ideal L2 Self	1					
2. Ought-to L2 Self	0.384 **	1				
3. PsyCap	0.400 **	0.342 **	1			
4. L2 Grit	0.409 **	0.137 **	0.178 **	1		
5. AI Literacy	0.127 **	0.051	0.049	0.049	1	
6. Engagement	0.399 **	0.240 **	0.530 **	0.365 **	0.338 **	1
M	3.814	3.411	3.584	3.339	3.602	3.496
SD	0.580	0.586	0.474	0.558	0.470	0.475
Skewness	−0.201	0.025	−0.027	−0.016	−0.060	0.113
Kurtosis	−0.137	−0.253	−0.116	−0.051	0.004	−0.024

** *p* < .01.

**Table 6:** Results of direct effects.

Path	B	β	SE	z	*p*
Ideal L2 self → PsyCap	0.258	0.316	0.024	10.645	***
Ideal L2 self → L2 grit	0.402	0.418	0.030	13.360	***
Ought-to L2 self → PsyCap	0.178	0.220	0.024	7.503	***
Ought-to L2 self → L2 grit	−0.022	−0.023	0.029	−0.776	0.438
Ideal L2 self → engagement	0.063	0.077	0.024	2.675	0.007
Ought-to L2 self → engagement	0.014	0.017	0.019	0.723	0.469
PsyCap → engagement	0.429	0.429	0.026	16.773	***
L2 grit → engagement	0.207	0.243	0.022	9.591	***
AI literacy → engagement	0.296	0.293	0.023	12.72	***

Note. B = unstandardized coefficient; β = standardized coefficient (STDYX). SE and z correspond to unstandardized coefficients. *** *p* < .001.

**Table 7:** Results of indirect effects.

Path	B	SE	95% BC CI		*p*
			LL	UL	
Ideal L2 self → PsyCap → Engagement	0.111	0.012	0.088	0.135	***
Ideal L2 self → L2 Grit → Engagement	0.083	0.011	0.063	0.106	***
Ought-to L2 self → PsyCap → Engagement	0.076	0.011	0.056	0.099	***
Ought-to L2 self → L2 Grit → Engagement	−0.005	0.006	−0.017	0.007	0.446

*** *p* < .001.

**Table 8:** Results of moderation effects.

**Interaction Effects**					
Path	B	SE	*p*	95% BC CI	
				LL	UL
PsyCap × AI Literacy → Engagement	0.180	0.053	.001	0.077	0.283
L2 Grit × AI Literacy → Engagement	0.161	0.045	***	0.074	0.253
**Simple Slopes: PsyCap → Engagement**					
AI Literacy Level	B	SE	*p*	LL	UL
Low (M − 1SD)	0.344	0.036	***	0.276	0.418
Mean (M)	0.429	0.026	***	0.379	0.479
High (M + 1SD)	0.513	0.035	***	0.443	0.578
**Simple Slopes: L2 Grit → Engagement**					
AI Literacy Level	B	SE	*p*	LL	UL
Low (M − 1SD)	0.131	0.030	***	0.074	0.190
Mean (M)	0.207	0.022	***	0.165	0.249
High (M + 1SD)	0.283	0.030	***	0.223	0.341

Note. B = unstandardized coefficient. SE corresponds to unstandardized coefficient. *** *p* < .001.

**Table 9:** Results of moderated mediation effects.

**Conditional Indirect Effects**						
Path	AI Literacy Level	B	SE	*p*	95% BC CI	
					LL	UL
Ideal L2 self → PsyCap → Engagement	Low (M − 1SD)	0.089	0.012	***	0.066	0.115
	Mean (M)	0.111	0.013	***	0.088	0.135
	High (M + 1SD)	0.132	0.016	***	0.104	0.166
Ideal L2 self → L2 Grit → Engagement	Low (M − 1SD)	0.053	0.013	***	0.029	0.080
	Mean (M)	0.083	0.011	***	0.064	0.107
	High (M + 1SD)	0.114	0.015	***	0.085	0.146
Ought-to L2 self → PsyCap → Engagement	Low (M − 1SD)	0.061	0.010	***	0.043	0.084
	Mean (M)	0.076	0.011	***	0.056	0.099
	High (M + 1SD)	0.092	0.013	***	0.067	0.120
Ought-to L2 self → L2 Grit → Engagement	Low (M − 1SD)	−0.003	0.004	0.463	−0.012	0.004
	Mean (M)	−0.005	0.006	0.448	−0.017	0.007
	High (M + 1SD)	−0.006	0.008	0.448	−0.024	0.010
**Index of moderated mediation (IMM)**						
Path	IMM	SE		*p*	95% BC CI	
					LL	UL
Ideal L2 self → PsyCap → Engagement	0.047	0.014		0.001	0.020	0.076
Ideal L2 self → L2 Grit → Engagement	0.065	0.019		0.001	0.028	0.104
Ought-to L2 self → PsyCap → Engagement	0.032	0.010		0.002	0.014	0.056
Ought-to L2 self → L2 Grit → Engagement	−0.004	0.005		0.479	−0.016	0.005

*** *p* < .001.

## Data Availability

The data presented in this study are available from the corresponding author upon reasonable request. They are not publicly available in order to protect participant confidentiality and to comply with the ethical requirements governing research involving human participants.
